# Association of Traffic-Related Air Pollution with Children’s Neurobehavioral Functions in Quanzhou, China

**DOI:** 10.1289/ehp.0800023

**Published:** 2009-05-11

**Authors:** Shunqin Wang, Jinliang Zhang, Xiaodong Zeng, Yimin Zeng, Shengchun Wang, Shuyun Chen

**Affiliations:** 1 Department of Occupational and Environmental Health Science, School of Public Health, Peking University, Beijing, People’s Republic of China; 2 Xiamen Municipal Health Bureau, Xiamen, Fujian Province, People’s Republic of China; 3 Division of Environmental Pollution and Health, Chinese Research Academy of Environmental Sciences, Beijing, People’s Republic of China; 4 Quanzhou Municipal Health Inspection Station; 5 Quanzhou Municipal Health Bureau and; 6 Licheng District Center for Disease Control and Prevention, Quanzhou, Fujian Province, People’s Republic of China

**Keywords:** air pollution, children, health effects, neurobehavioral functions, traffic-related air pollution

## Abstract

**Background:**

With the increase of motor vehicles, ambient air pollution related to traffic exhaust has become an important environmental issue in China. Because of their fast growth and development, children are more susceptible to ambient air pollution exposure. Many chemicals from traffic exhaust, such as carbon monoxide, nitrogen dioxide, and lead, have been reported to show adverse effects on neurobehavioral functions. Several studies in China have suggested that traffic exhaust might affect neurobehavioral functions of adults who have occupational traffic exhaust exposure. However, few data have been reported on the effects on neurobehavioral function in children.

**Objectives:**

The objective of this study was to explore the association between traffic-related air pollution exposure and its effects on neurobehavioral function in children.

**Methods:**

This field study was conducted in Quanzhou, China, where two primary schools were chosen based on traffic density and monitoring data of ambient air pollutants. School A was located in a clear area and school B in a polluted area. We monitored NO_2_ and particulate matter with aerodynamic diameter ≤ 10 μm as indicators for traffic-related air pollution on the campuses and in classrooms for 2 consecutive days in May 2005. The children from second grade (8–9 years of age) and third grade (9–10 years of age) of the two schools (*n* = 928) participated in a questionnaire survey and manual-assisted neurobehavioral testing. We selected 282 third-grade children (school A, 136; school B, 146) to participate in computer-assisted neurobehavioral testing. We conducted the fieldwork between May and June 2005. We used data from 861 participants (school A, 431; school B, 430) with manual neurobehavioral testing and from all participants with computerized testing for data analyses.

**Results:**

Media concentrations of NO_2_ in school A and school B campus were 7 μg/m^3^ and 36 μg/m^3^, respectively (*p* < 0.05). The ordinal logistic regression analyses showed that, after controlling the potential confounding factors, participants living in the polluted area showed poor performance on all testing; differences in results for six of nine tests (66.7%) achieved statistical significance: Visual Simple Reaction Time with preferred hand and with nonpreferred hand, Continuous Performance, Digit Symbol, Pursuit Aiming, and Sign Register.

**Conclusion:**

We found a significant relationship between chronic low-level traffic-related air pollution exposure and neurobehavioral function in exposed children. More studies are needed to explore the effects of traffic exhaust on neurobehavioral function and development.

Traffic-related air pollution—basically urban outdoor pollution—is a global public health issue. With the increase in motor vehicles, traffic exhaust has become one of the major sources of ambient air pollution in many large- and medium-sized cities, and air pollution related to traffic exhaust has become an important environmental issue in China. Environmental monitoring data showed that with the rise of vehicle traffic density, vehicle pollution on busy roads in many cities of China has become a serious problem, with concentrations of nitrogen dioxide and carbon monoxide significantly violating the national standard. Therefore, adverse health effects of vehicle exhaust have gained increasing attention from scientists throughout China as well as around the world. Studies have shown that main health effects of traffic-related air pollution include irritation effects as well as effects on the respiratory and cardiovascular systems. Irritation induced by photochemical smog affects the eye, nose, pharynx, larynx, trachea, and lung (Cheng et al. 2003). Reported adverse health effects on the respiratory system include higher prevalence of reported respiratory symptoms, such as chronic cough and expectoration (Nitta et al. 1993), dyspnea, bronchitis, rhinitis (Van et al. 1997), and lower lung function. Gauderman et al. (2007) found that children who lived within 500 m of a freeway (motorway) had substantial deficits in 8-year growth of forced expiratory volume in 1 sec (−81 mL) and maximum mid-expiratory flow rate (−127 mL/sec, *p* = 0.03), compared with children who lived at least 1,500 m from a freeway. Regarding effects on cardiovascular system, since the study by Peters et al. (2004) on myocardial infarction in Augsburg, in southern Germany, more articles on the effects of traffic exhaust on cardiovascular system have been published. Evidence indicates that ambient particulate matter (PM) could have adverse effects on the cardiovascular system (Chen and Guo 2005).

Although the cardiorespiratory effects and mechanisms have been more thoroughly investigated, little is known about the possible neurologic effects. Calderon-Garciduenas et al. (2002) found that dogs living in a highly polluted region in Mexico City had increased brain inflammation compared with those living in a less polluted area. Another study found that the brain tissue of mice in Mexico City had higher levels of nuclear factor-κB activation and nitric oxide production, as well as the principal proinflammatory cytokines interleukin (IL)-1 and tumor necrosis factor (TNF)-α, compared with those from nonpolluted areas (Campbell et al. 2005). Donaldson et al. (2005) reported that ultrafine carbon particles have been found in the olfactory bulb and the cerebrum and cerebellum in rats after inhalation exposure. Calderon-Garciduenas et al. (2004) found that exposure to severe air pollution has been associated with the increased cyclooxygenase-2, an inflammatory mediator, and accumulation of the 42-amino-acid form of amyloid beta protein, a cause of neuronal dysfunction in humans. Margai and Henry (2003) reported that long-term traffic pollution exposure may affect human learning ability, coordination, memory, and judgment. Kilburn (2000) demonstrated slowness of response, memory loss, and disordered sleep suggestive of neurobehavioral impairment in workers whose occupations involved significant indoor diesel exhaust exposure. They also detected abnormalities such as visual field defects, delayed blink reflex latency, and balance impairment, as well as impaired recall memory, problem solving, and perceptual motor speed tests. Tang et al. (2008) found an association between prenatal polycyclic aromatic hydrocarbon (PAH) exposure and neurodevelopment at 2 years of age.

Compared with adults, children grow and develop quickly, and thus are considered especially susceptible to neurotoxicants because of *a*) greater external exposures due to play activities on or near streets, *b*) greater external exposures per unit of body weight, and *c*) greater vulnerability of the developing central nervous system to neurotoxicants. Therefore, more researchers are studying children. Some studies have reported the adverse health effects of traffic exhaust on children’s neurobehavioral functions. Perera et al. (2006) collected PAHs in particulate form with individual pumps during 2 consecutive days in 181 pregnant women from New York City (New York, USA), and measured the mental health of their offspring with the Bayley Scales of Infant Development at 3 years of age. Their results suggested that PAH exposure at levels recently encountered in New York City ambient air might adversely affect children’s cognitive development. Effects of prenatal exposure to PAHs were also explored in China (Tang et al. 2008). Levels of PAH–DNA adducts and developmental quotients (DQs) in motor, adaptive, language, and social areas were measured in a cohort of non-smoking women and their newborns enrolled between March 2002 and June 2002. The results showed that decrements in one or more DQs were significantly associated with cord blood levels of PAH–DNA adducts (Tang et al. 2008). Another study examined the relation between black carbon and cognition among 202 children in a prospective birth cohort study at Boston, Massachusetts (USA; 1986–2001) (Suglia et al. 2008). After adjustment for possible confounding factors (sociodemographic factors, birth weight, blood lead level, and tobacco smoke exposure), black carbon was associated with decreases in the vocabulary, matrix, and composite IQ scores of the Kaufman Brief Intelligence Test and with decreases on the visual subscale and general index of the Wide Range Assessment of Memory and Learning. Higher levels of black carbon predicted decreased cognitive function across assessments of verbal and nonverbal intelligence and memory constructs (Suglia et al. 2008).

Other studies in China have examined neurobehavioral function and occupational traffic exhaust exposure. Liu et al. (2001) tested 166 traffic policemen (exposed group) and 164 non-traffic policemen (control group) with the World Health Organization (WHO) Neurobehavioral Core Test Battery (NCTB) in Kunming City (Yunnan Province). The selected tests included Mood States, Simple Reaction Time (SRT), Digit Span (DSP), Santa Ana Manual Dexterity (SAMD), Digit Symbol (DSB), Benton Visual Retention (VRT), and Pursuit Aiming (PAT) tests. The results showed that the scores of DSP, SAMD, and PAT in the exposed group were significantly lower than those of the control group (47.99 ± 10.59, 47.96 ± 10.08, and 48.29 ± 8.82 vs. 50.35 ± 9.72, 50.51 ± 10.50, and 51.28 ± 10.73, respectively). Very similar results were also reported by Liu and Zhong (1994). After testing the neurobehavioral function of 202 traffic policemen and 171 nontraffic policemen by WHO NCTB, they found that the exposed group had lower scores in DSP, VRT, DSB, and PAT. The longer the length of service years as traffic policemen, the lower the NCTB scores. Wang et al. (2002) also used the NCTB to test neurobehavioral function of the selected 70 male drivers and 52 undergraduate students. The scores of all items for drivers were lower than those for the students. However, in the literature we did not find any reports on traffic exhaust and children’s neurobehavioral function in China. Our previous study (unpublished data) found that children living in areas polluted with high levels of traffic exhaust showed lower scores in Continuous Performance (CPT) and in Line Discrimination than those living in a lightly polluted area. However, scientific conclusions cannot be drawn because of the limitations of sample size and comparability of subjects’ sociodemographic characteristics. In the present study, we used the new testing battery with fewer computerized neurobehavioral testing items than before and added manual neurobehavioral testing, which made it possible to increase the sample size. This gave us more opportunity to control for potential confounding factors.

## Materials and Methods

### Study area selection

**Figure 1:**
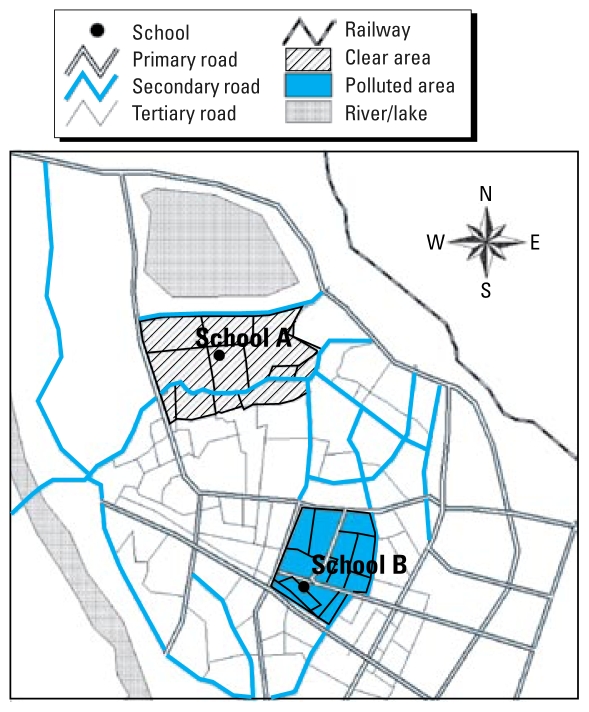
Study areas, including clear area and polluted area, in Quanzhou (Fujian Province, China). The polluted area is located in the center of Quanzhou with many primary (arterial, with heaviest traffic) roads, whereas the clear area is located in the north of Quanzhou with few primary roads, about 20 km away from polluted area. School A was in a clear area about 3.5 km away from primary roads, and school B was in the polluted area located on a three-way intersection of primary roads with heavy traffic.

We identified study areas based on ambient air quality monitoring data (2002) for Quanzhou, Fujian Province, China, and local traffic density. The polluted area is located in the center of Quanzhou, which has many arterial roads with high traffic density and traffic congestion. The buildings in this area are high and closely packed, which may hamper the spread of pollutants. The average annual concentrations of NO_2_ and PM_10_ in 2002 were 22 μg/m^3^ and 80 μg/m^3^, respectively. The clear area (lightly polluted) is located in the north of Quanzhou city and has few arterial roads and low traffic density, about 20 km away from the polluted area, and has few high buildings. Average annual concentrations of NO_2_ and PM_10_ in 2002 were 7 μg/m^3^ and 68 μg/m^3^, respectively. There are no industrial or other sources of local air pollution around these two areas, nor is any industrial pollution likely to be transported to either site from other areas or from outside the city. We selected one primary school in each area to conduct a field study. School A, in the clear area, is located about 3.5 km away from primary roads (arterial roads) and has low traffic density (1,528 cars/hr in nonpeak traffic time, 15 August 2004). School B, in the polluted area, is located on a three-way intersection of primary roads (arterial roads) with heavy traffic and long detention time, during which traffic can slow or even stop (6,740 cars/hr in nonpeak traffic time, 15August 2004) (Figure 1).

### Ambient air quality monitoring at schools

On 24 and 25 May 2005, we measured the levels of ambient air NO_2_ and PM_10_ (including lead in PM_10_) at each school as indicators of traffic-related air pollution. We located five monitoring sites for NO_2_ in each primary school: one at the school gate, two on the playground, and two in classrooms; we located two sites for ambient air PM_10_ and lead concentration in each school, at the school gate and on the playground. We collected 24-hr–averaged NO_2_ concentrations by passive samplers provided by the Chinese Center for Disease Prevention and Control, determined by the Saltzman method (Song et al. 1994). We conducted the assay in the laboratory within 72 hr after 24-hr continuous sampling. We set the sampling head at a height of 2.0 m to keep it out of children’s reach. PM_10_ samplers were at a height of 1.2 m. These samplers were analyzed for PM_10_ and lead by Quanzhou Environmental Monitoring (Quanzhou, China), a professional institution for environmental monitoring, by weighing and by graphite furnace atomic absorption spectrophotometry, respectively. All monitoring work lasted for 2 days, and we used 2-day–averaged values because of limitations of time and funds.

### Identification and selection of participants

Fieldwork was conducted between May and June of 2005. We selected children from second grade (8–9 years of age) and third grade (9–10 years of age) from two schools (*n* = 861; school A, 431; school B, 430) to participate in a questionnaire survey and manual-assisted neurobehavioral testing. Informed consent forms were signed by the parents of all children who participated in the survey. The eligibility criteria for selecting students for data analyses were as follows: *a*) length of residence ≥ 3 years and place of residence ≤ 5 km away from their school, *b*) no reported psychoses or neurologic hereditary diseases in the children or their family, *c*) good health during testing time, *d*) questionnaire completed, and *e*) permission to participate in this study given by the parents or guardians. From these children, we also selected 282 students from three third-grade classes (school A, 136; school B, 146) to participate in computerized tests.

### Questionnaire survey

Self-reporting questionnaires with informed consent were handed out to all second-grade and third-grade students by their head teachers and filled out by their parents or guardians. An introductory letter was included with the questionnaires that informed parents of the main goals and activities of the project and noted that they had the right to decline participation of their child if they so desired. We also informed them that they did not have to answer every question on the questionnaire. Only children whose parents or guardians permitted their participation were included in this study. The questionnaire included such questions as parental and child’s medical history, mother’s pregnancy history, parental occupational histories, education years, socioeconomic status, tobacco and alcohol consumption, child’s residential history, type of heating, and child’s living condition.

### Neurobehavioral testing

We chose nine standardized tests as the test battery to assess neurobehavioral performance for four neurologic functions: cognitive, motor, sensory, and psychomotor (Table 1). The testing was finished between 27 May and 5 June 2005. The test battery consisted of five computerized tests chosen from the Neurobehavioral Evaluation System (NES; Chinese version, 2000) (Tan et al. 2003) and four manual tests from the Jinyi Psychomotor Test Battery (Wang et al. 2000). All testing was conducted in a separate classroom guided by trained investigators. All investigators were blinded to study aims and children’s exposure groups. They used the standard oral instructions and made sure that children fully understood the testing process. Furthermore, no children were given the real test until all children passed the simulated pre-test. The children were instructed to answer questions and ask for assistance if necessary during testing.

### Computerized testing

#### Line Discrimination (LDT)

A longer line (165 × 2 pixels) and a shorter line (155 × 2 pixels) were displayed randomly on the computer screen. The children were instructed to observe carefully and press the spacebar on the keyboard as quickly as possible when they saw the longer line displayed on the screen. A total of 70 lines (the target line appearing seven times) were displayed with the stimulus duration and interval both 1 sec.

#### Visual Retention (VRT)

A geometric diagram was displayed on the computer screen, and children were instructed to keep its shape and characteristic in mind. Then the diagram was replaced by a display of four diagrams, numbered 1, 2, 3, and 4, one of which was the target diagram. Children were instructed to press the number key corresponding to the target diagram, as quickly as possible. A total of 15 trials were administered with the stimulus duration of 4 sec.

#### Visual Simple Reaction Time (VSRT)

A red square was displayed 20 times on the computer screen, with a stimulus duration of 2 sec. Children were asked to press the spacebar on the keyboard with their preferred hand (Prh) as soon as they saw the red square on the screen. Then the trial was repeated with the nonpreferred hand (Nprh).

#### Continuous Performance (CPT)

Large letters were flashed briefly on the computer screen, and children were asked to press the return key on the keyboard as quickly as possible when they saw the letter “S,” which appeared 30 times intermittently in the sequence of 60 letters. The stimulus duration was 700 msec, and each stimulus was replaced on the screen instantaneously with the next one.

### Manual testing

#### Digit Erase (DET)

The children were presented with a paper with many digits on it and were instructed to erase the digits as quickly as possible in the order stated by examiners. The trial lasted for 2.5 min, and then the numbers of correct erasings were recorded.

#### Digit Symbol (DSB)

Children were each given a sheet of paper with a grid that paired each of nine different symbols with one digit, from 1 to 9. A similar grid was presented on another sheet, with digits 1–9 in serial order and blank spaces for the corresponding symbols. The children were instructed to write down the matching symbol for each digit as quickly as possible. The time for the trial was 90 sec. One correct pairing was scored as 1. The total scores (total numbers of correct pairings) were recorded.

#### Pursuit Aiming (PAT)

The children were presented with a paper with small circles on it and were instructed to add a dot to the center of each circle as quickly as possible, in the direction shown on the paper. The numbers of correct dottings were recorded.

#### Sign Register (SRT)

The children were presented with a paper with many squares on it and were instructed to enter the radical of the Chinese character 
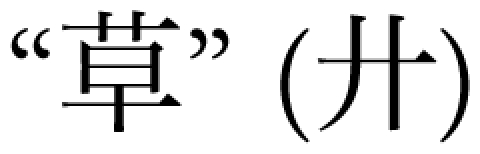
 in the squares as quickly as possible, in the direction showed on the paper. The numbers of correct entries were recorded.

### Data analyses

#### Scoring the tests

For manual neurobehavioral testing, scores are expressed as the numbers of correct responses; for the computerized neurobehavioral testing, scores are expressed as Neurobehavioral Ability Index (NAI), which is an integrated index directly computed by NES with the following formula (Xu et al. 2001):


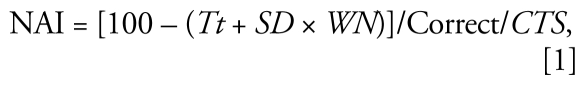


where *Tt* is the total time (in seconds), *SD* is the standard deviation of the correction coefficient (0.116 sec/time), *WN* is the number of wrong performance times, Correct is the number of correct performance times, and *CTS* is the SD of average time of correct performance (sec/time).

We entered all data into a single computer file and conducted statistical analysis using SPSS software, version 12.0 (SPSS Inc., Chicago, IL, USA). We used one-way analyses of variance (for large sample size) and Mann–Whitney *U*-tests (for small sample size) for continuous variables, and chi-square tests for comparing categorical variables between two groups. We performed ordinal logistic regression analyses to assess the magnitude and nature of the associations among neurobehavioral outcomes or if key factors for the scores or NAI and their log-transformed values were not normally distributed. Moreover, multivariate linear regression diagnostics indicated that some linear regression assumptions were not met. Therefore, we used ordinal logistic regression instead of multivariate linear regression analyses to examine the association of traffic exhaust exposure and other factors with the children’s neurobehavioral function. First, we converted the NAI or testing scores to ordered categorical variables in four levels (excellent, good, not bad, and bad), each corresponding to a quartile of distribution of the NAI or testing score. We then used a proportional odds cumulative logit model to examine the association between neurobehavioral outcomes and 13 independent variables: age (years), body mass index (BMI), educational attainment of children’s father (0 = college diploma, 1 = senior middle school or vocational school, 2 = junior middle school, 3 = elementary school or illiteracy), exposure status (0 = clear area, 1 = polluted area), sex (0 = male, 1 = female), birth weight (0 = ≥ 2,500 g, 1 = < 2,500 g), delivery method (0 = vaginal delivery, 1 = abdominal delivery), passive smoking (0 = no, 1 = yes), open kitchen (separate from sitting room) (0 = no, 1 = yes), familiarity with computer games (0 = no or little, 1 = much), household fuel (0 = coal, 1 = other), vision (0 = normal, 1 = poor), and breast-feeding (0 = ≥ 6 months, 1 = < 6 months). We identified these independent variables based on other studies on neurobehavioral functions (Otto et al. 1996; Sunyer 2008; Walkowiak et al. 1998) and our univariate analyses.

## Results

### Air pollution estimates

**Figure 2:**
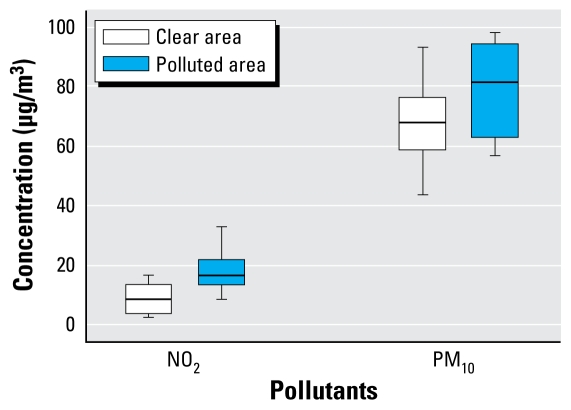
Data from daily monitoring of ambient air quality by Quanzhou Environmental Monitoring Station in 2002. The polluted area had higher concentrations of NO_2_ and PM_10_. Horizontal line is median; box indicates 25th–75th percentile; error bars indicate maximum and minimum.

Monthly air monitoring data of Quanzhou in 2002 showed that the average concentrations of NO_2_, and PM_10_ were, respectively, 22 μg/m^3^ and 80 μg/m^3^ in the polluted area and 7 μg/m^3^ and 68 μg/m^3^ in the clear area. The Mann–Whitney *U*-test (two-sided, α = 0.05) showed that there was significant difference for NO_2_ between the polluted area and the clear area but no significance for PM_10_ (*U* = 1.647, *p* = 0.101; Figure 2).

Median NO_2_ concentrations at schools A and B were 7 μg/m^3^ and 36 μg/m^3^, respectively, which are consistent with the data from the environmental monitoring state in 2002. A Mann–Whitney *U*-test indicated that these were significantly different (*U* = 2.627, *p* = 0.008). However, differences in levels of PM_10_ and lead were not significant (Table 2).

### Sociodemographic characteristics of participants

Self-report questionnaires were handed out to 963 students, and 928 were returned. Among 928 students, 67 were excluded from manual testing (39 from school A and 28 from school B) for the following reasons: parents or guardians did not grant permission to participate in the study (16 children), place of residence > 5 km away from their school (21 children), length of residence < 3 years (15 children), invalid questionnaire responses (13 children), history of epilepsy (1 child), and hemorrhinia (nosebleed) at testing time (1 child). Overall, 861 children (431 in school A, 430 in school B) who finished manual testing and 282 who finished both manual and computerized testing (136 in school A, 146 in school B) were eligible for data analyses.

Table 3 compares characteristics for participants who entered the data analyses with those excluded. More children were excluded in school A (12.8%) than in school B (8.3%); no other compared characteristics showed significant difference, which suggested that excluding some participants did not result in confounding in this study.

For the 861 children who participated in the manual neurobehavioral testing, the two groups had similar distribution for sex, familiarity with computer games, passive smoking, breast-feeding, birth weight, and ventilation device in kitchen. Although children from school A had significantly lower height and body weight than those from school B, their BMI showed no significant difference. However, children from school B reported a higher percentage of poor vision, and their fathers had achieved higher education, compared with those from school A. For both groups, a minority of participants reported that their families used coal for cooking (3.73%) and heating (1.62%), and we found no significant differences between the two groups in these factors. Average distance between children’s households and their schools was 1.96 ± 1.19 km, and 88.4% of them lived within 3 km from their schools. The children from school B lived a little farther from school than those from school A. In addition, all children involved in this study had lived in their current households > 3 years. Of them, 65.6% had lived there > 5 years. The average durations were 5.63 ± 2.52 years for school A and 5.89 ± 2.41 years for school B (*p* = 0.120).

For the 282 children who finished both manual and computerized testing, those from school A and school B were almost the same age and had similar distributions for sex, familiarity with computer games, delivery method, household fuel, type of winter heating, birth weight, and use of open kitchen. However, children from school B were taller than those from school A. Compared with children from school A, a higher proportion of children from school B reported passive smoking exposure. The children from school A reported 5.76 ± 2.6 years’ length of residence, whereas those from school B reported 6.29 ± 2.47 years (Table 4).

### Neurobehavioral testing results

Children from school A had higher scores or NAI values in all items for testing. Analysis of variance (ANOVA) showed significant differences for scores for DSB, PAT, and SRT and for the CPT NAI (*p* < 0.05) (Table 5).

Table 6 presents the models of ordinal logistic regression analyses. After controlling the potential confounding factors, 11 covariates were significantly associated with the outcome, especially age and exposure status. Age was shown in seven models, which indicated that being older was associated with better performance on the seven tests. Girls obtained higher scores for CPT and DET than did boys. Passive smoking was a key factor for poor performance on VRT and DSB, and coal burning at home was significantly associated with poorer performance on DET. Children with normal vision had better performance on CPT than did those with poor vision. Higher BMI was associated with higher NAI of LDT, and lower birth weight was associated lower NAI of VRT and PAT. More important, the results showed that children in the polluted area had poorer performance on all tests, and six children (66.7%) achieved statistical significance. All results suggested that exposure to air pollution related to traffic exhaust was significantly associated with the decrease in NAI/scores of VSRT-Prh (*p* = 0.044), VSRT-Nprh (*p* = 0.017), CPT (*p* < 0.001), DSB (*p* = 0.019), PAT (*p* < 0.001), and SRT (*p* < 0.001).

## Discussion

### Effects of vehicle exhaust on neurobehavioral function and its mechanism

Many chemicals that form vehicle exhaust, such as CO, nitrogen oxides, and lead, have adverse effects on neurobehavioral functions (Zhang et al. 2005), and possible potentiation or synergy among pollutants aggravates the effect to some degree. So far, epidemiologic studies on the effects of vehicle exhaust on neurobehavioral function have focused on occupational populations, such as traffic policemen and bus conductors. Only a few studies have been conducted with children. The present study showed that children in a school in a polluted area (school B) had lower scores in all items for testing than did those from a school in a clear area (school A). After adjusting the potential confounders, we found significant associations between traffic-related air pollution exposure and poorer performance on VRT-Prh, VRT-Nprh, CPT, DSB, PAT, and SRT neurobehavioral tests, which measure children’s psychomotor, motor, and sensory functions. The results indicate that motor vehicle–related ambient air pollution may have a negative impact on children’s neurobehavioral function, which agrees with results of two recent studies (Perera et al. 2006; Suglia et al 2008). However, we found no significant difference for VRT (which was measured to assess participants’ cognition) between the two groups, whereas Perera et al. (2006) and Suglia et al. (2008) found an association between cognition and traffic-related air pollution. This difference may relate to the small sample size in our study. Therefore, it is necessary to explore the association of traffic exhaust with children’s neurobehavioral function in future research.

Several potential mechanisms could explain the associations found in this study. Chronic respiratory tract inflammation may lead to brain inflammation by altering levels of circulating cytokines, such as TNF-α and IL-1. These cytokines have the ability to up-regulate cyclooxygenase-2, a potent active mediator of inflammation, in capillary brain endothelium (Campbell 2004). An alternative hypothetical mechanism for the neurologic effect of air pollution is based on the observation that metals in ultrafine PM are able to translocate directly to the brain, without entering the lung (Calderon-Garciduenas et al. 2002). These are all hypotheses, and more work in this area remains to be done.

### Selection of study areas and study subjects

The areas and subjects chosen for scientific study play a vital role in environmental epidemiology, especially with ecologic study designs. Subjects should have different levels of exposure to the target factor (in this study, traffic-related air pollution) and comparable levels of exposure to all possible confounding factors. In this study, these requirements have been well satisfied. Environment monitoring data showed that concentrations of NO_2_ in the polluted area were significantly higher than those in the clear area. The differences can be attributed to the different levels of vehicle exhaust because there were no local industrial pollution sources around the two schools and no decoration in the classroom or households. Unfortunately, PM_10_ levels at two schools were similar, which did not accord with the monitoring results of NO_2_ levels, although it is in accordance with the data reported by the environmental monitor station. We explain this as follows: *a*) The two schools may also be polluted by some large-diameter PM from other sources, such as dust from construction, landfills, and household fuel burning. *b*) Traffic-related PM was mainly PM_2.5_ (aerodynamic diameter ≤ 2.5 μm) and the weight of PM_2.5_ is low, which would not contribute much to PM_10_. Lead levels at the two schools were also similar; leaded gasoline has been prohibited since 2000, and perhaps lead in the two areas derived not from traffic pollution, but rather from such sources as building material pollution or secondary pollution. In addition, other sociodemographic characteristics of children from the two primary school are highly comparable: *a*) Both of the two schools are the central primary school of their district and have comparable teaching levels according to information from the local education authority. *b*) The children have similar living conditions and life habits. *c*) Types of household fuel and heating in winter are comparable. Questionnaire surveys showed that only 3.6% of the households use coal as household fuel; 67.36% have no heating in winter, and only 1.62% households use heating in winter and coal as well. Statistical tests indicated that there was no significant difference for the type of fuels and the way for heating between the two schools. *d*) Children attended the primary schools by geographic district in Quanzhou, which guaranteed that the children in the same school lived in the same atmospheric environment. The questionnaire survey showed that the average distance between children’s households and their school was 1.96 ± 1.19 km and 88.4% children lived within 3 km from their school. More important, all children involved in this study lived in their current household > 3 years, and 58.7% lived there > 5 years. The average duration was 5.63 ± 2.52 years for the children in school A and 5.89 ± 2.41 years for school B (*p* = 0.120), which indicates that this finding in our study is an effect of chronic exposure to traffic exhaust. All these suggest that the study areas and subjects we selected in our study meet the study design.

### Application of the neurobehavioral function testing battery

We used two main types of neurobehavioral testing: manual neurobehavioral testing and computerized neurobehavioral testing. Computerized testing is an effective method to evaluate neurobehavioral function and is now widely used around the world. The advantage of computerized testing is that it guarantees standardization of testing regimens, efficiency of data collection, computerization of data storage and analyses, and systematization of testing administration. However, some of the computerized tests are not suitable for young children and people with fewer years of education. At the same time, the performances on computerized tests are highly associated with children’s computer experience (Williamson 1996). Although it falls behind in standardization, systematization, and accuracy, manual testing can be easily administered and is less sensitive to cultural background. In this study, we used both manual and computerized testing to complement each other’s disadvantages and comprehensively evaluate children’s neurobehavioral function. To minimize the impact of computer familiarity on the results of computerized testing, we applied “one-key performance” in our study, which means that children completed the test by pressing one particular key only. The findings of manual testing were consistent with those of computerized testing. Therefore, we concluded that manual neurobehavioral testing is useful in developing countries. The other advantage of manual testing is that it can be conducted for several subjects, which makes it possible to study large-sample epidemiology.

### Indicators for traffic-related air pollution

It is known that traffic exhaust includes many complex chemicals. Therefore, we are not able to assess the adverse effects uniquely associated with single agents. In this case, the adverse effects observed in this study might be considered the consequences of complex mixture exposure. In this study, we used NO_2_ and PM_10_ as indicators for traffic-related air pollution. Through the measurement of ambient NO_2_ and PM_10_ concentrations and the routine environmental monitoring data, we assessed the children’s air pollution exposure levels. The results from the study by Rijnders et al. (2001) showed that personal exposure and outdoor NO_2_ levels were significantly influenced by the degree of urbanization of the city district and by the traffic density and the distance to a nearby highway. They concluded that NO_2_ might be considered as the indicator of traffic-related air pollution. PM_2.5_ was also considered as an indicator for traffic-related air pollution in many other studies (Hoek et al. 2002; Suglia et al. 2008). Unfortunately, PM_2.5_ was not included in daily air quality monitoring in Quanzhou, and we were not able to measure the PM_2.5_ levels at the schools at that time because of limitations of equipment and funds. However, our study suggested that NO_2_ but not PM_10_ can be a good indicator for the traffic-related air pollution in China.

### The limitations of the study

Limitations of this study are as follows: *a*) We had few data available on ambient air pollution in Quanzhou. Although 1-year local air quality monitoring data from the Quanzhou environmental monitoring station was available for traffic-related air pollution in the study areas, we could collect only 2 days of school air quality monitoring data. Therefore, we could not estimate seasonal differences in air pollution and its effects on children’s neurobehavior. *b*) To assure the comparability of the two groups and to control for confounding, we selected two similar schools for study, which would affect the generalizability of the results. *c*) Of the children in the schools, 92.8% were allowed to participate the study. We could not get more information to check the comparability between participants and nonparticipants because of no questionnaires were returned from nonparticipants. *d*) The sample size for computerized neurobehavioral function testing was relatively small due to time and other limitations during the fieldwork, which might leave more residual confounding compared with the manual testing in our study. *e*) This is a cross-sectional, epidemiologic study with an ecologic design, and we selected only two exposure groups to study due to limitations of time and funds. Therefore, inferences of causality are not possible, and further study is needed to confirm the results. Moreover, we considered only 13 covariates in the study, and many other factors may have been ignored that may also contribute some residual confounding. However, we could not estimate the effect of residual confounding because we did not conduct sensitivity analyses in this study.

In conclusion, the results of this study suggest that there is a significant association between traffic-related air pollution exposure and poorer performance on neurobehavioral testing of urban, community-dwelling, school-age children, which indicates that chronic low-level exposure to traffic-related air pollution might have a negative impact on children’s neurobehavioral function. This study also suggests that it is important that more studies explore the potentially neurotoxic effects of traffic-related air pollution in order to control motor vehicle pollution and maintain children’s health.

## Figures and Tables

**Table 1:** Neurobehavioral test battery used in this study.

Name of test	Function	Domain
Computer-assisted testing		
Line Discrimination (LDT)	Visual perception—vigilance and attention	Sensory
Visual Retention (VRT)	Visual memory	Cognition
Visual Simple Reaction Time, preferred hand (VRT-Prh)	Visual response speed	Psychomotor
Visual Simple Reaction Time, nonpreferred hand (VRT-Nprh)	Visual response speed	Psychomotor
Continuous Performance (CPT)	Psychomotor—speed and attention	Psychomotor

Manual testing		
Digit Erase (DET)	Visual detecting speed and accuracy	Psychomotor
Digit Symbol (DSB)	Visual perception—accuracy and speed	Sensory
Pursuit Aiming (PAT)	Psychomotor stability	Psychomotor
Sign Register (SRT)	Motor coordination	Motor

**Table 2:** Results of air pollutants monitored for exposure estimates (μg/m^3^).

	School A			School B		
Pollutant	Playground	Classroom	School gate	Playground	Classroom	School gate
NO_2_	8	8	7	37	28	36
PM_10_	68	—	67	81	—	89
Lead	0.12	—	0.14	0.13	—	0.14

**Table 3:** Comparison of characteristics for participants included in the data analyses and those excluded (data from school roster and school physical examination in April 2005).

Variable	Participants	Nonparticipants	*p*-Value
Age [years (mean ± SD)]	9.09 ± 0.73	9.23 ± 0.80	0.077
Height [cm (mean ± SD)]	133.96 ± 7.68	132.75 ± 7.50	0.137
Weight [kg (mean ± SD)]	30.97 ± 8.05	30.71 ± 8.17	0.768
BMI (mean ± SD)	17.23 ± 4.18	17.36 ± 3.92	0.732
School [no. (%)]			
School A	431 (87.2)	63 (12.8)	
School B	430 (91.7)	39 (8.3)	0.016
Sex [no. (%)]			
Male	471 (90.6)	49 (9.4)	
Female	390 (88.0)	53 (12.0)	0.121
Vision [no. (%)]			
Normal	688 (90.2)	75 (9.8)	
Poor	173 (87.4)	25 (12.6)	0.154

**Table 4:** Sociodemographic characteristics of participants in school A and school B.

	Manual testing			Computerized testing		
Characteristic	School A	School B	*p*-Value	School A	School B	*p*-Value
Age [years (mean ± SD)]	9.09 ± 0.75	9.09 ± 0.72	0.96	9.67 ± 0.51	9.60 ± 0.41	0.22
Height [cm (mean ± SD)]	133.00 ± 7.71	135.17 ± 7.46	< 0.001	135.36 ± 8.19	138.03 ± 6.64	0.003
Weight [kg (mean ± SD)]	30.10 ± 7.72	31.92 ± 8.26	0.001	32.00 ± 9.22	33.95 ± 9.06	0.074
BMI (mean ± SD)	17.00 ± 4.31	17.43 ± 4.03	0.174	17.51 ± 5.35	17.76 ± 4.37	0.674
Length of residency [years (mean ± SD)]	5.63 ± 2.52	5.89 ± 2.41	0.12	5.76 ± 2.60	6.29 ± 2.47	0.084
Distance of residence from school [km (mean ± SD)]	1.71 ± 1.13	2.21 ± 1.19	< 0.001	1.81 ± 1.28	2.63 ± 1.30	< 0.001
Sex [no. (%)]						
Male	239 (55.45)	232 (53.95)		78 (57.35)	71 (48.63)	
Female	192 (44.55)	198 (46.05)	0.659	58 (42.65)	75 (51.37)	0.143
Vision [no. (%)]^a^						
Normal	363 (84.22)	325 (75.58)		111 (81.62)	105 (71.92)	
Poor	68 (15.78)	105 (24.42)	0.002	25 (18.38)	41 (28.08)	0.055
Familiarity with computer games [no. (%)]^b^						
Much	229 (55.72)	260 (61.32)		92 (69.17)	107 (73.79)	
None or little	182 (44.28)	164 (38.68)	0.1	41 (30.83)	38 (26.21)	0.394
Educational attainment of participants’ fathers [no. (%)]						
≤ Primary school	33 (7.91)	21 (4.98)		10 (7.63)	6 (4.11)	
Middle school	158 (37.89)	104 (24.64)		54 (41.22)	24 (16.44)	
High school or technical school	167 (40.05)	188 (44.55)		57 (43.51)	70 (47.95)	
≥ College/university	59 (14.15)	109 (25.83)	< 0.001	10 (7.63)	46 (31.51)	< 0.001
Second-hand smoke [no. (%)]^c^						
Yes	247 (58.53)	235 (55.29)		80 (60.61)	67 (45.89)	
No	175 (41.47)	190 (44.71)	0.342	52 (39.39)	79 (54.11)	0.014
Delivery method [no. (%)]						
Vaginal delivery	343 (82.65)	303 (71.97)		105 (80.77)	103 (72.54)	
Abdominal delivery	72 (17.35)	118 (28.03)	< 0.001	25 (19.23)	39 (27.46)	0.11
Breast-feeding [no. (%)]						
≥ 6 months	367 (85.15)	360 (83.72)		117 (86.03)	121 (82.88)	
< 6 months	64 (14.85)	70 (16.28)	0.563	19 (13.97)	25 (17.12)	0.466
Household fuel [no. (%)]						
Coal	19 (4.40)	12 (2.80)		4 (2.99)	2 (1.36)	
Others	412 (95.60)	418 (97.20)	0.203	130 (97.01)	144 (98.64)	0.361
Winter heating [no. (%)]						
No heating	297 (68.91)	283 (65.81)		96 (70.59)	92 (63.01)	
Air conditioning	125 (29.00)	142 (33.02)	0.278	40 (29.41)	54 (36.99)	0.178
Coal burning	9 (2.09)	5 (1.16)				
Ventilation device in kitchen [no. (%)]						
Yes	354 (82.13)	352 (81.86)		117 (86.03)	121 (82.88)	
No	77 (17.87)	78 (18.14)	0.917	19 (13.97)	25 (17.12)	0.466
Birth weight [no. (%)]						
≥ 2,500 g	403 (94.60)	402 (93.71)		129 (94.85)	132 (90.41)	
< 2,500 g	23 (5.40)	27 (6.29)	0.577	7 (5.15)	14 (9.59)	0.156
Open kitchen [no. (%)]						
Yes	71 (17.40)	43 (10.36)		22 (17.05)	15 (10.71)	
No	337 (82.60)	372 (89.64)	0.003	107 (82.95)	125 (89.29)	0.131

a Poor vision includes such conditions as myopia, hypermetropia, astigmatism, and anopsia.

b Playing games ≥ 2 times every week identified as “much.”

c One or both parents smoke in the home.

**Table 5:** The results of ANOVA.

	School A		School B			
Tests	No.	Mean ± SD	No.	Mean ± SD	*F*-test	*p*-Value
Computerized testing (NAI)						
LDT	133	0.24 ± 0.08	141	0.24 ± 0.10	0.182	0.670
VRT	135	0.65 ± 0.19	146	0.65 ± 0.20	0.017	0.897
VSRT-Prh	135	11.75 ± 6.48	143	10.68 ± 6.54	1.878	0.172
VSRT-Nprh	135	9.78 ± 5.72	143	8.68 ± 5.73	2.533	0.113
CPT	133	0.91 ± 0.08	146	0.87 ± 0.14	10.257	0.002
Manual testing (score)						
DET	423	60.96 ± 17.32	429	58.65 ± 19.85	1.805	0.071
DSB	415	41.62 ± 12.79	423	38.53 ± 9.96	3.902	< 0.001
PAT	426	95.52 ± 35.80	430	84.27 ± 27.83	5.138	< 0.001
SRT	425	42.34 ± 15.58	430	37.40 ± 11.62	5.261	< 0.001

**Table 6:** The model of ordinal logistic regression analyses.

	LDT		VRT		VSRT-Prh		VSRT-Nprh		CPT		DET		DSB		PAT		SRT	
Variable	OR	*p*-Value	OR	*p*-Value	OR	*p*-Value	OR	*p*-Value	OR	*p*-Value	OR	*p*-Value	OR	*p*-Value	OR	*p*-Value	OR	*p*-Value
Age	0.56	0.018	0.85	0.561	0.36	< 0.001	0.51	0.013	0.90	0.687	0.46	< 0.001	0.63	< 0.001	0.80	0.020	0.74	0.001
BMI	0.96	0.094	1.10	0.001	1.03	0.209	1.00	0.997	0.99	0.553	1.03	0.060	1.01	0.534	0.98	0.217	0.98	0.143
Educational attainment of subjects’ fathers																		
x = 1	1.00	0.997	1.10	0.875	0.82	0.720	1.58	0.424	0.64	0.429	1.32	0.391	0.85	0.606	0.58	0.088	0.62	0.134
x = 2	1.61	0.157	0.43	0.027	0.53	0.097	0.68	0.296	0.49	0.057	0.93	0.727	0.75	0.147	0.88	0.529	0.61	0.012
x = 3	1.38	0.270	0.63	0.161	0.97	0.915	0.90	0.754	0.94	0.835	1.07	0.724	0.80	0.217	0.89	0.534	0.60	0.005
Exposure status	1.12	0.624	1.61	0.069	1.67	0.044	1.83	0.017	2.40	0.001	1.25	0.102	1.38	0.019	1.64	< 0.001	1.94	< 0.001
Sex	0.71	0.116	1.58	0.057	1.12	0.645	1.25	0.347	0.39	< 0.001	0.67	0.003	1.15	0.291	1.12	0.401	1.06	0.663
Birth weight	1.31	0.497	2.78	0.035	1.29	0.573	1.17	0.730	0.80	0.614	0.79	0.418	1.03	0.915	1.75	0.049	1.02	0.957
Delivery method	0.90	0.696	0.46	0.010	1.87	0.031	1.26	0.414	1.15	0.619	1.15	0.375	1.07	0.668	1.02	0.887	1.00	0.981
Second-hand smoke	0.83	0.388	1.72	0.027	1.10	0.684	1.50	0.090	0.83	0.439	1.22	0.144	1.32	0.040	1.02	0.889	1.11	0.448
Open kitchen	0.83	0.557	1.13	0.741	1.18	0.638	1.54	0.221	0.83	0.611	0.81	0.292	0.89	0.581	1.03	0.887	1.11	0.607
Household fuel	3.53	0.070	0.36	0.200	1.76	0.463	2.26	0.305	4.64	0.074	0.46	0.029	1.44	0.302	1.49	0.249	1.09	0.795
Familiarity with computer games	0.89	0.620	0.57	0.041	0.70	0.193	1.11	0.700	0.82	0.453	0.68	0.007	1.20	0.200	1.08	0.580	1.10	0.510
Vision	1.17	0.545	0.61	0.085	0.98	0.937	0.86	0.599	1.78	0.042	1.13	0.476	1.26	0.172	1.18	0.337	1.36	0.067
Breast-feeding	1.61	0.124	1.26	0.512	1.31	0.433	1.58	0.187	1.05	0.887	1.14	0.480	0.87	0.455	0.90	0.582	1.11	0.584

ORs represent the association between 1 unit increment in the variable and decrement in one or more grade(s) of neurobehavioral testing (four grades: excellent, good, not bad, bad).
